# Resveratrol Ameliorates Brain Functions During Gestation in Mancozeb-Low-Dose-Induced Brain Disorders via Mitigating Inflammation, Lipid Peroxidation, Mitochondrial Dysfunction, DNA Damage, Cell Death, and Histopathological Alterations

**DOI:** 10.1007/s12035-026-06167-2

**Published:** 2026-09-23

**Authors:** Rana M. El-Sawah, Amoura M. Abou-El-Naga, Nermeen E. Ashry, Heba A. El-Ghaweet

**Affiliations:** Department of Zoology, Faculty of Science, Mansoura University, Mansoura, 35516 Egypt

**Keywords:** Brain disorders, Mancozeb, Resveratrol, DNA damage, Hippocampus, Frontal cerebral cortex

## Abstract

Worldwide, many old and young people suffer from neurodegenerative brain disorders. Continuous exposure to mancozeb (MNZ), a fungicide extensively used worldwide on a wide range of crops, is a serious threat to public health because of inducing neuroinflammation and oxidative stress. Resveratrol (RES) is a natural polyphenol with antioxidant and anti-inflammatory effects. This study evaluated the neuroprotective potential of RES against MNZ low-dose-induced brain damage during gestation and elucidated its underlying mechanisms. Thirty-two pregnant Wistar rats were randomly allocated into four groups (*n* = 8): control, RES-treated rats (100 mg/kg/day, intraperitoneal injection), MNZ-treated rats (100 mg/kg/day, oral), and RES + MNZ-treated rats (100 mg/kg/day intraperitoneal before 100 mg/kg/day MNZ oral). Treatments were administered daily from gestational day (GD) 1 to GD 20. On GD 20, brain samples were collected for biochemical, DNA integrity, morphometric, histopathological, and immunohistochemical assays. Gestational MNZ exposure induced neuroinflammation, lipid peroxidation, reduced CAT activity, and triggered GFAP accumulation, resulting in astrogliosis in the cerebrum and hippocampus. Furthermore, MNZ disrupted mitochondrial apoptosis regulatory proteins, increased DNA damage in maternal and fetal brain cells, induced cerebral and hippocampal neurodegeneration, and reduced cortical thickness while increasing the hippocampal stratum lucidum thickness. Remarkably, pretreatment with RES protected the maternal and fetal brains, preserving cerebral and hippocampal-dependent brain functions through suppressing lipid peroxidation, inflammation, mitochondrial-mediated apoptosis, DNA damage, and histopathological changes. In conclusion, RES demonstrates a potent prophylactic neuroprotective efficacy during gestation against MNZ-induced neurotoxicity, suggesting its potential value as a preventive dietary agent against environmental toxin-mediated brain disorders.

## Introduction

Neurodegenerative diseases are expected to be a serious challenge for medicine in the coming years. Neuron degeneration is mainly responsible for serious mental disorders and neuropsychiatric diseases. Neurodegenerative disorders have several pathogenic mechanisms for the progression of the diseases, so understanding the underlying mechanism is important to resist neurodegeneration [1].

Although safeguarding crops from pests is a critical issue, there is strong evidence that the widespread use of agricultural pesticides in the environment contributes to the global silent pandemic of neurodevelopmental disorders. Exposure of mothers to broad-spectrum agricultural pesticides, such as pyrethroids like deltamethrin and carbamates like MNZ, has been shown to affect embryonic brain cytoarchitecture, cause oxidative stress, and disrupt neurodevelopment. At low levels of exposure, many environmental toxins can pass easily through the placental blood into the fetal blood, causing permanent brain damage that would have minimal effect on adults. The developing brain in the sensitive periods of intrauterine development, infancy, and early childhood is highly susceptible to toxic chemical exposure [2].

Mancozeb (MNZ) is a manganese-containing fungicide that is used globally on many different crops and is associated with severe neurodegenerative disorders [3, 4]. MNZ elevates oxidative stress in brain tissues through manganese ions (Mn^2+^) accumulation [4]. The chronic neurodegeneration can be accelerated by MNZ, leading to neurodegenerative disorders [5]. MNZ promotes cell apoptosis and mitochondrial malfunction in the brain by releasing Mn^2+^, which causes high oxidative stress and mitochondrial alterations in the brain cells [4].

Recently, alternative medicine involving plant-derived medicine has gained growing interest [6]. Polyphenols such as resveratrol (RES) have been mentioned in recent years as protective and therapeutic agents to suppress the progression of neurological diseases through their antioxidant and anti-inflammatory effects [7]. RES is derived from several plants, such as the seeds and skin of grapes, and its neuroprotective, antioxidant, and anti-inflammatory capabilities have attracted wide interest recently [8]. RES has promising therapeutic potential against neurodegenerative diseases [9].

RES enhances brain functions by activating silent information regulator-1 (SIRT1). SIRT1 plays a crucial role in the development of neurons, preventing oxidative stress and neuronal apoptosis [10]. RES maintains mitochondrial integrity and enhances motor and cognitive disorders in patients with Parkinson's disease [11]. RES enhances DNA stability by promoting DNA repair pathways [12]. RES maintains the redox balance and promotes the antioxidant system in neurons [13].

However, the information on the MNZ-low dose effects on the brain during the sensitive gestation period has not been fully clarified. Along with that, the underlying mechanisms of MNZ neuronal damage in pregnancy have not been completely investigated yet. On the other hand, RES is a potential neuroprotective agent even though information on the ameliorating mechanisms against MNZ low-dose-induced gestational brain disorders is insufficient. Therefore, we aimed to study the potential neuro-prophylactic effects of RES and its distinct mechanisms against the brain damage induced by MNZ low dose in pregnant rats.

We hypothesized that RES would protect against gestational MNZ exposure neurodegeneration because of its strong antioxidant, anti-inflammatory, and antiapoptotic properties. To test our hypothesis, we targeted the NF-κB, TNF-α, and iNOS signaling pathways as well as neuroglial activation (GFAP expression), lipid peroxidation, antioxidant defenses, mitochondrial apoptotic pathway, DNA damage in mother and fetus, histopathological alterations in hippocampal main regions and frontal cerebral cortex, as well as the correlation and relationships between all these parameters. Understanding these connections could result in new protective strategies to stop or postpone neurological issues linked to MNZ gestational exposure.

## Methods

### Chemicals

Mancozeb (MNZ) (CAS No.: 8018-01−7) and trans-resveratrol (RES) (CAS No.: 501-36−0) were obtained from Sigma-Aldrich Company (St. Louis, MO, USA). All other chemicals and reagents were of the highest quality and analytical grade.

### Animals and Ethical Approval

Mature male and female Wistar rats weighing 150 ± 5 g were provided by the VACSERA Company, Cairo, Egypt. For two weeks, all rats were allowed to acclimate to standard laboratory conditions at a constant temperature of 24 ± 2 °C on a 12/12-h alternating light/dark cycle with free access to standard rat chow and water ad libitum. Regular cleaning and sterilization of the cages with 70% ethanol were performed. The biosafety procedures during animal treatment, euthanasia, and management with biological tissue samples were followed according to the methods described earlier by McCormick-Ell and Connell [14]. This research followed the ethical principles adopted by the Mansoura University Animal Care and Use Committee (MU-ACUC) (NIH publication No. 86–23, revised 1985). All research and animal welfare were approved by MU-ACUC, approval number: MU-ACUC (SC.MS.24.11.81).

### Experimental Design

This study is a complementary study to our previous study [15] to focus deeply on the mitigating effects of RES against the specific regional brain injuries induced by MNZ.

After acclimatization, the female rats were randomly isolated in clean cages for mating with healthy males. Overnight, each male rat was paired with two female rats with a normal estrus cycle. The next morning, the vaginal smears were collected and stained with methylene blue, and then the presence of sperm was investigated [16]. The day after the positive test for sperm observation in the smear was considered GD1.

**Fig. 1:**
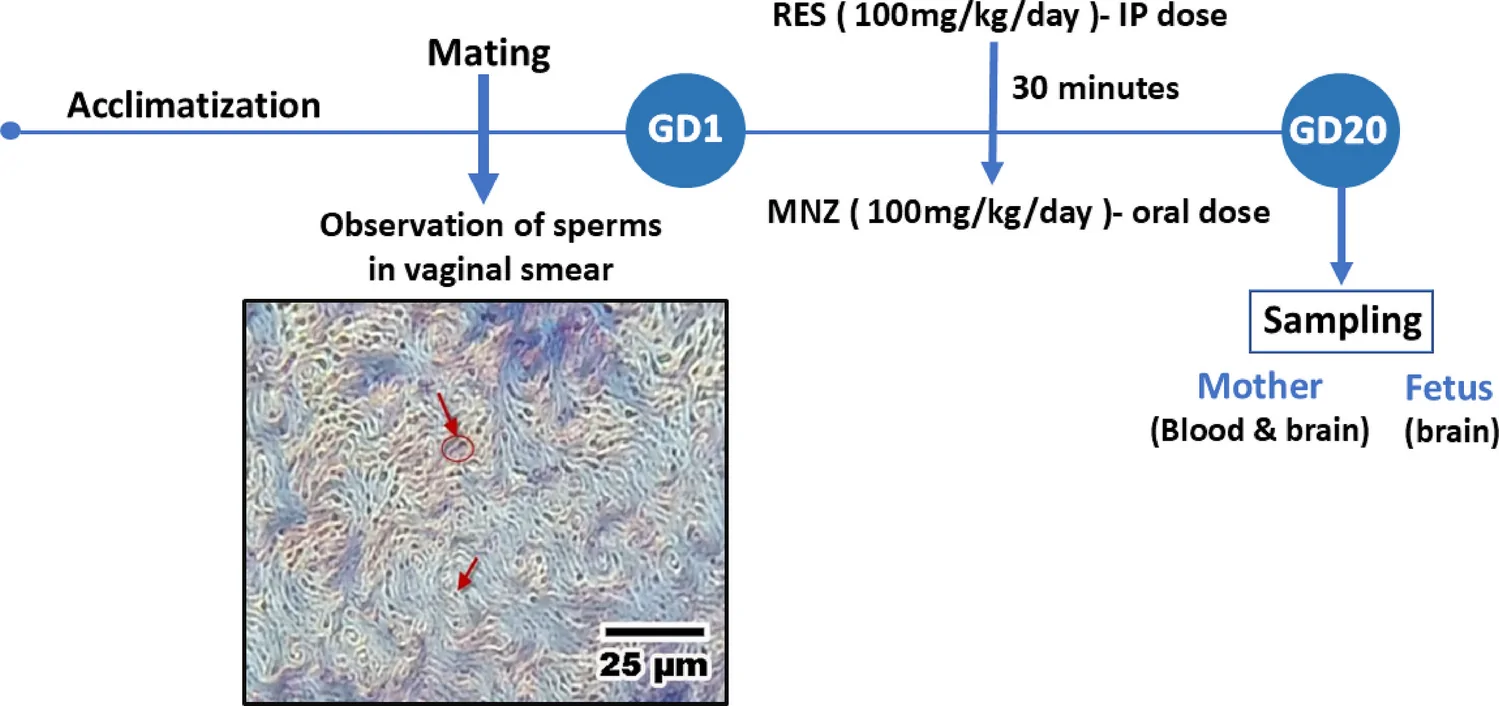
Graphical illustration of the experimental design. Timeline included 2-week acclimatization followed by overnight mating (2 females:1 male). The day after observation of sperm (red arrows) in vaginal smears stained with methylene blue was considered gestational day 1 (GD1). The pregnant rats were randomly distributed into 4 groups (*n*=8). The treatments continued for 20 days fromGD1 to GD20.The control did not receive any treatment. RES rats were intraperitoneally injected with 100 mg/kg/day RES. MNZ rats were orally administered 100 mg/kg/day MNZ. RES+MNZ group was orally administered MNZ daily after 30 minutes of daily intraperitoneal RES injection. On GD20, the pregnant rats were anesthetized after an overnight fast, then blood samples were collected from mother rats, followed by euthanasia under anesthesia, and then maternal and fetal brains were collected. RES, Resveratrol; MNZ, Mancozeb; IP, intraperitoneal

After mating, the pregnant rats were randomly divided into four groups of eight rats each. All the treatments were performed from GD1 to GD20. The control group did not receive any treatment. The RES group was intraperitoneally injected with RES, 100 mg/kg body weight [17] daily for 20 days. Each day for 20 days, the MNZ group received MNZ orally by gastric tube, 100 mg/kg/day, that considered a low dose [18]. For 20 days, the RES + MNZ group received an intraperitoneal injection of RES 30 min before a daily oral dose of MNZ (Fig. 1). Pregnant rats were weighed daily to record weight changes for adjusting the appropriate doses of RES and MNZ.

### Sample Collection

On gestational day 20, the pregnant rats were weighed and anesthetized with a mixture of xylazine and ketamine (5 mg and 80 mg per kg body weight, respectively) by intraperitoneal injection after an overnight fast [19]. Blood samples were drawn directly from the heart by cardiac puncture into sterilized serum-separator tubes, followed by centrifugation for 10 min at 3000 rpm. The sera were properly separated into clean tubes, labeled, and kept at −20 °C until biochemical analysis. Rats were euthanized by cervical dislocation under anesthesia. The brains of mothers and their fetuses were collected. The maternal brains were separated and homogenized in 50 mM cold potassium phosphate buffer pH 7.4, then centrifuged at 5000 rpm for 5 min at 4 °C in a refrigerated centrifuge. The supernatant was separated, labeled, and stored at −20 °C for biochemical investigations.

### Morphometric Assessments

During the dissection, the weight and length changes in the brain and cerebellum of mothers and fetuses were reported. Moreover, the cerebellum: brain weight ratio was calculated. The relative weights (%) were calculated using this formula: Relative organ weight (%) = (organ weight (g)/ body weight (g))× 100, as stated by Hamid et al. [20]. Euthanized rats were disposed of using the alkaline hydrolysis method according to Alderman et al. [21].

### Biochemical Analyses

Sera concentrations of inflammatory cytokines, tumor necrosis factor alpha (TNF-α; pg/mL) and nuclear factor kappa B (NF-κB; ng/mL) were analyzed by the sandwich enzyme immunoassay technique according to the manufacturers' protocols of ELISA kits (Catalog # CSB-E11987r and MBS453975) provided by Cusabio (Wuhan, China) and MyBioSource (San Diego, California, USA), respectively. The color intensity was measured at 450 nm.

A rat ELISA kit obtained from Biodiagnostic company (Dokki, Giza, Egypt) was used to estimate the concentrations of lipid peroxidation end-product malondialdehyde (MDA; nmol/g, Catalog # MD 25 29) in the brain by colorimetric assay in accordance with the manufacturer’s instructions. The thiobarbituric acid reactive substances (TBARS) assay is based on the interaction of MDA with thiobarbituric acid (TBA) in an acidic solution for 30 min at 95 °C, which forms a thiobarbituric acid reactive compound. This reaction creates a pink colored MDA-TBA adduct that can be detected spectrophotometrically at 534 nm.

In brain homogenate, the activity of antioxidant enzyme catalase (CAT; U/g) was estimated colorimetrically by an ELISA kit (Catalog # CA 25 17, Biodiagnostic company, Dokki, Giza, Egypt). One unit of CAT decomposes 1 µM of H_2_O_2_ per minute into water and oxygen. Then, horseradish peroxidase catalyzes the reaction of the undecomposed H_2_O_2_ with 4-aminophenazone and 3,5-dichloro-2-hydroxybenzene sulfonic acid, resulting in the formation of a chromophore. Then, the absorbance was measured spectrophotometrically at 510 nm ± 10 nm. Because the amount of color produced is entirely dependent on how much H_2_O_2_ was left over, the color intensity is inversely proportional to the CAT level in the brain sample.

The quantitative estimation of the levels of Bcl-2-associated X protein (Bax; ng/g tissue) and B-cell lymphoma 2 (Bcl-2; ng/g tissue) in the brain tissue homogenate was performed using the protocol of sandwich rat ELISA kits following the manufacturer's instructions (Bax, Catalog # RTFI00370) and (Bcl-2, Catalog # RTEB0450) from AssayGenie (South-East Inner City, Dublin, Ireland). The optical density was measured at 450 nm.

### Single-Cell Gel Electrophoresis (Comet Assay)

Using the comet assay method described by Singh et al. [22], the DNA damage in the brains of the mother rats at GD20 and their 20-day-old fetuses was determined. After homogenization using an automatic homogenizer (0.024 M Na_2_EDTA, 0.075 M NaCl, pH 7.5), centrifugation using a cooling centrifuge (1500 rpm for 10 min at 0 °C) was performed. Then, frosted slides were layered twice with 100 µL 1% GP-42 agarose; 75 µL of supernatant were mixed with 75 µL of 2% LGT agarose, the slide was covered and left to solidify, and 100 µl of agarose GP-42 1% was layered on the surface and covered with another slide and allowed to gel. Then, the slides were soaked in 100mM Na_4_EDTA, 2.5 M NaCl, 10 mM Tris base, 1% sarcosinate, Triton X-100, and 10% dimethyl sulfoxide at 4 °C for 1–24 h in the dark to lyse cell membranes and strip away proteins, leaving only the DNA bound within nucleoids. Then, electrophoresis was conducted (25 V, 300 mA, 1 mM Na_2_EDTA, and 300 mM NaOH, pH 13) for 20 min in the dark, neutralization in 400 mM Tris buffer (pH 7.5) for 7 min, dehydration in ethanol for 5 min, and staining dry slides with ethidium bromide. Examination was performed with a fluorescent microscope with a green filter. The Komet 5 image analysis software randomly selected 100 cells per sample to analyze the DNA migration length (μm), the migrated DNA percentage (%), and the tail moment estimated through multiplying the tail length by the migrated DNA percentage.

### Histopathological Investigations

Brain tissue samples were fixed in 10% neutral buffered formalin (pH 7.4). After washing with water, dehydration with an ethanol ascending series, clearing with xylene, and embedding in paraffin wax were performed, respectively. Following that, the specimens were oriented and cut into 5-µm sections using a rotary microtome. Consequently, the sections were mounted on the slides and then stained with hematoxylin and eosin [23]. Histopathological examination of the brain sections was carried out using a bright-field Olympus light microscope, and the photographs were captured with an Olympus camera. Then, the histomorphometric investigations of photographs were accomplished by using ImageJ.

### Immunohistochemical Assessment of iNOS and GFAP

In accordance with the guidelines of Al-Garni et al. and Luijerink et al. [24, 25], deparaffinized brain sections were rehydrated before the microwave antigen retrieval process was applied. Afterward, the endogenous peroxidase activity was quenched with 3% H_2_O_2_, CH_3_OH, and phosphate-buffered saline solution and then blocked with 10% normal horse serum. Following the manufacturer’s protocol, overnight incubation was conducted at room temperature with primary antibodies, involving anti-iNOS rabbit polyclonal antibody (Catalog # ab15323; Abcam, Cambridge, UK; 1:100 Dilution) and GFAP mouse monoclonal antibody (Catalog # 901-065−052423; Biocare Medical, Pacheco, California, USA; 1:100 Dilution). Subsequently, the secondary antibody and Avidin–Biotin Complex (ABC) were added, then the slides were stained with 3,3′-diaminobenzidine (DAB) to produce the brown color, and the slides were counterstained with Meyer’s hematoxylin, producing the blue color and providing high contrast against the brown DAB stain. Examination and photography of the slides were performed using an Olympus digital camera and an Olympus microscope. The quantification of the immunoreactive areas in the resulting images was carried out using the ImageJ program.

### Statistical Analysis

The data were represented as the means ± standard deviation (SD) (*n* = 6). Using SPSS software version 27.0, the statistical comparisons were conducted by one-way analysis of variance (ANOVA) followed by Tukey’s multiple comparison post hoc test at 0.05, 0.01, and 0.001 levels. Significance was presented as follows: ^*^P < 0.05, ^**^P < 0.01, and ^***^P < 0.001 versus the control. ^&^*P* < 0.05, ^&&^*P* < 0.01, and ^&&&^*P* < 0.001 versus the MNZ group. GraphPad Prism 8.0 software was used for data visualization as graphs. OriginPro 2024 software was used to perform a polar heatmap of correlation and a correlation matrix.

## Results

### RES Ameliorated Brain Morphometry in MNZ-Treated Rats and Their Fetuses

**Fig. 2:**
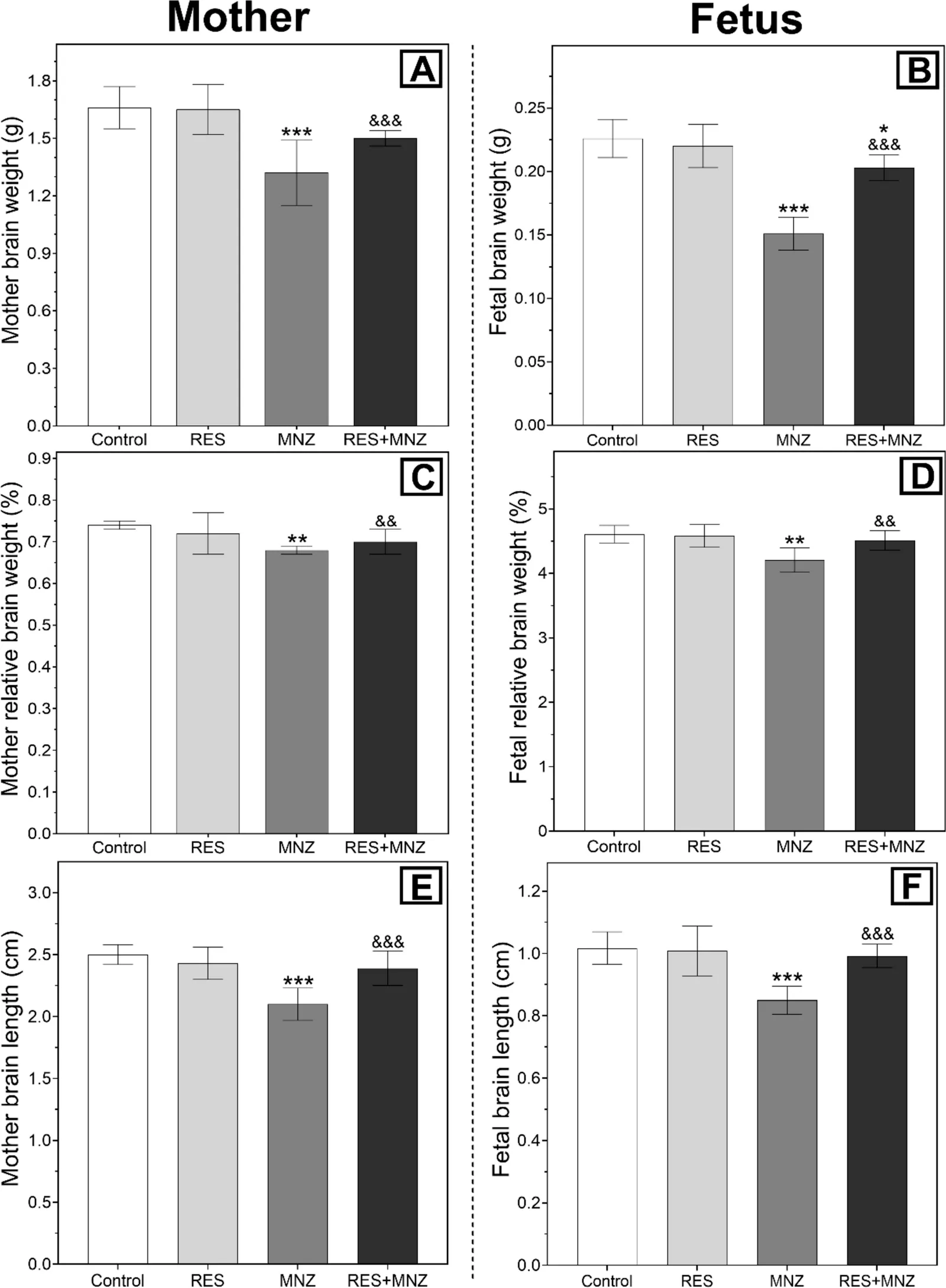
Effect of RES and MNZ on the maternal brain weight (g; **A**), the weight of fetal brain (g; **B**), relative weight of mother's brain (%; **C**), fetal relative brain weight (%; **D**), adult brain length (cm; **E**), and length of fetus' brain (cm; **F**). Data are means ± SD (*n* = 6); ^*^P < 0.05, ^**^P < 0.01, and ^***^P < 0.001 versus the control; ^&&^*P* < 0.01, ^&&&^*P* < 0.001 compared to the MNZ group according to Tukey’s test. RES, Resveratrol; MNZ, Mancozeb

As brain morphometric measurements play a notable role in brain diseases, maternal and fetal brain morphometric findings were evaluated in Fig. 2. MNZ administration caused a significant (*P* < 0.001 and *P* < 0.01) reduction in the lengths, weights, and relative weights of brains in adults (−16%, −20.48%, and −8.11%, respectively) and fetuses (−16.42%, −33.19%, and −8.7%, respectively) as compared with the control. Interestingly, RES mitigated these reductions (*P* < 0.001 and *P* < 0.01) when injected before MNZ treatment as compared with the MNZ group (mother: 13.81%, 13.64%, and 2.94%, respectively; fetus: 16.71%, 34.44%, and 7.28%, respectively). Meanwhile, administration of RES alone caused nonsignificant changes in brain morphometric parameters when compared to control.

### RES Mitigated Inflammatory Mediators in MNZ-Administered Pregnant Rats

**Fig. 3:**
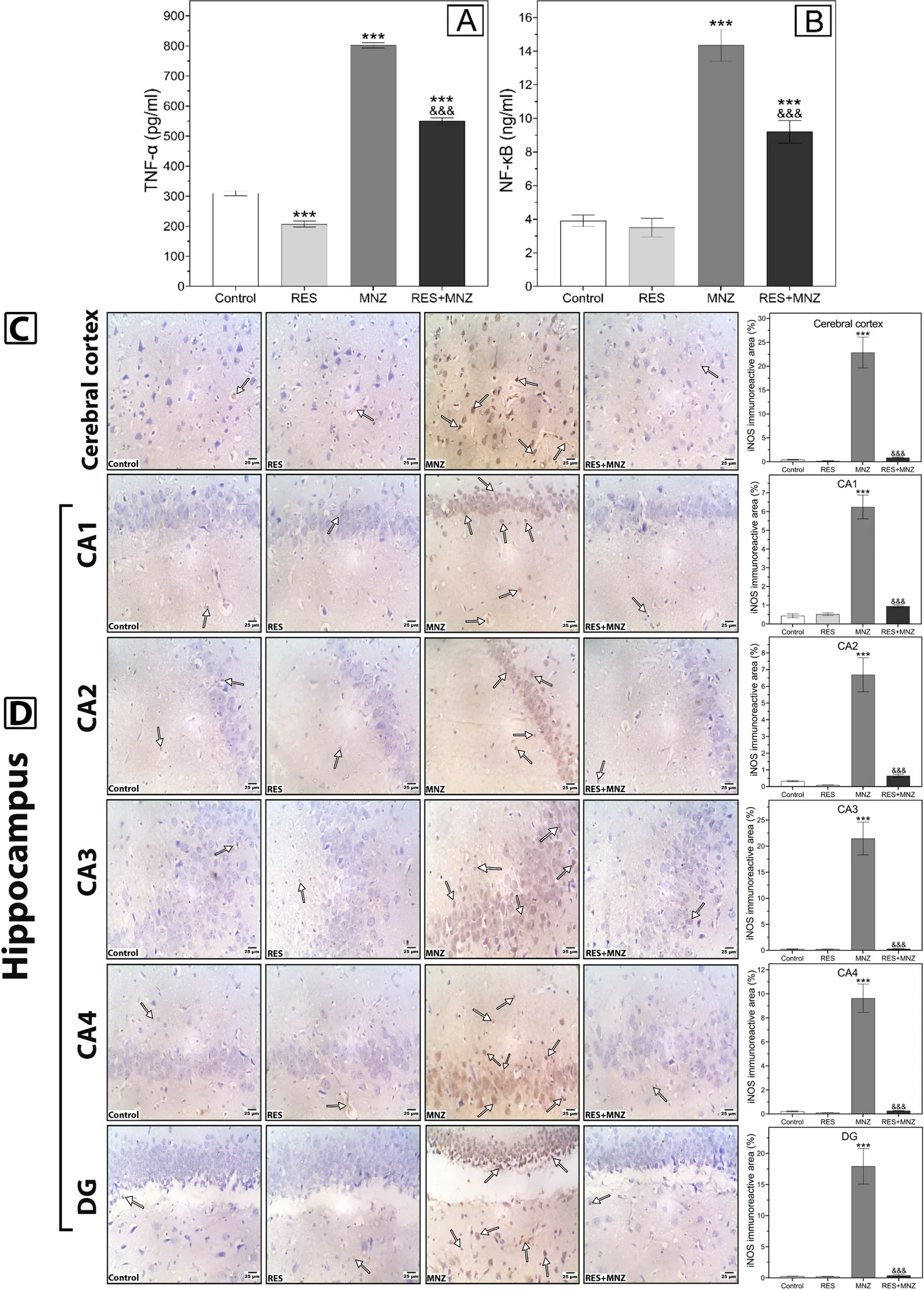
Effect of RES and MNZ on the serum levels of TNF-α (pg/mL; **A**), NF-κB (ng/mL; **B**), and immunohistochemical expression of iNOS in cerebral cortex (**C**), hippocampus, including Cornu Ammonis (CA1, CA2, CA3, and CA4), and dentate gyrus (DG) regions (**D**) in the control group and other experimental pregnant rat groups (X400). Values of iNOS expression levels are shown as means ± SD of 6 microscopic fields/tissue sample of iNOS immunoreactive area %. The control and RES groups show a slight expression of iNOS in the sections of the cerebral cortex and hippocampus (arrow). MNZ-treated rats exhibit marked and severe expression of iNOS in cerebral and hippocampal sections (arrows). RES + MNZ group reveals an almost normal iNOS expression as compared to the control in all the various brain sections (arrow). Data are means ± SD (*n* = 6); ^***^P < 0.001 versus the control; ^&&&^*P* < 0.001 compared to the MNZ group according to Tukey’s test. RES, resveratrol; MNZ, mancozeb

Since inflammatory cytokines play a crucial role in the pathogenesis of brain diseases, their levels were analyzed as shown in Fig. 3. MNZ-treated rats showed a significant (*P* < 0.001) increase in TNF-α (158.71%) and NF-κB (267.95%) serum levels along with iNOS expression in the cerebral cortex and hippocampus (CA1, CA2, CA3, CA4, and DG) compared with the control. In contrast, RES dramatically (*P* < 0.001) reduced these elevated cytokine levels (-−31.42% and −35.89%, respectively) and iNOS expression in cerebral and hippocampal tissues in the RES + MNZ group compared with the MNZ group. Meanwhile, RES injected alone significantly (*P* < 0.001) decreased TNF-α levels (−32.9%) compared with the control and kept NF-κB levels in serum, as well as iNOS expression in brain tissues at the control level.

### RES Controlled Lipid Peroxidation and Modulated CAT Activity in the Brains of MNZ-Treated Rats

**Fig. 4:**
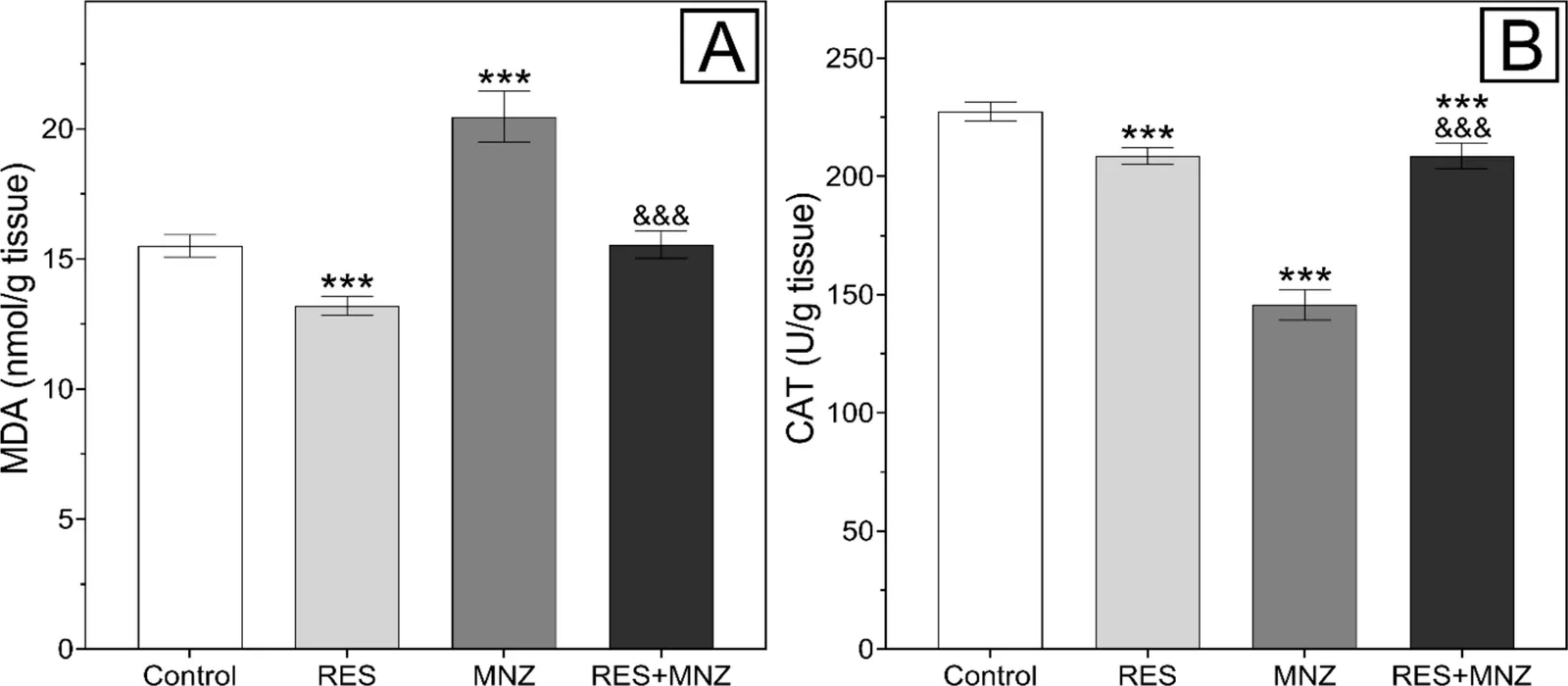
Effect of RES and MNZ on MDA level (nmol/g tissue; **A**) and CAT activity (U/g tissue; **B**) in the brain tissues of the control and other studied pregnant rat groups. Data are represented as means ± SD (*n* = 6); ^***^, ^&&&^ indicate statistical significance at *P* < 0.001; ^***^ is compared to the control; ^&&&^ is versus the MNZ group according to Tukey’s test. RES, resveratrol; MNZ, mancozeb

The preventive effect of RES against lipid peroxidation in brain tissues of pregnant rats counteracting the MNZ-induced effects was displayed in Fig. 4. The MNZ increased lipid peroxidation through the dramatic elevation in MDA levels in the brain tissue in MNZ-treated rats (32.06%, *P* < 0.001), while CAT activity in the brain notably decreased (−35.95%, *P* < 0.001) when compared with the control. On the other hand, RES + MNZ-treated rats showed a significant reduction in MDA levels in the brain (−24.04%, *P* < 0.001), whereas CAT activity in brain tissue was significantly improved compared with MNZ-treated rats (43.22%, *P* < 0.001). In comparison to the control, RES alone injection significantly reduced MDA in the brain (−14.84%, *P* < 0.001). However, the CAT activity in the RES group was lower (−8.29%, *P* < 0.001) than that of the control.

### RES Protected Against Astrogliosis by Modulating GFAP Expression in the Brains of MNZ-Treated Rats

**Fig. 5:**
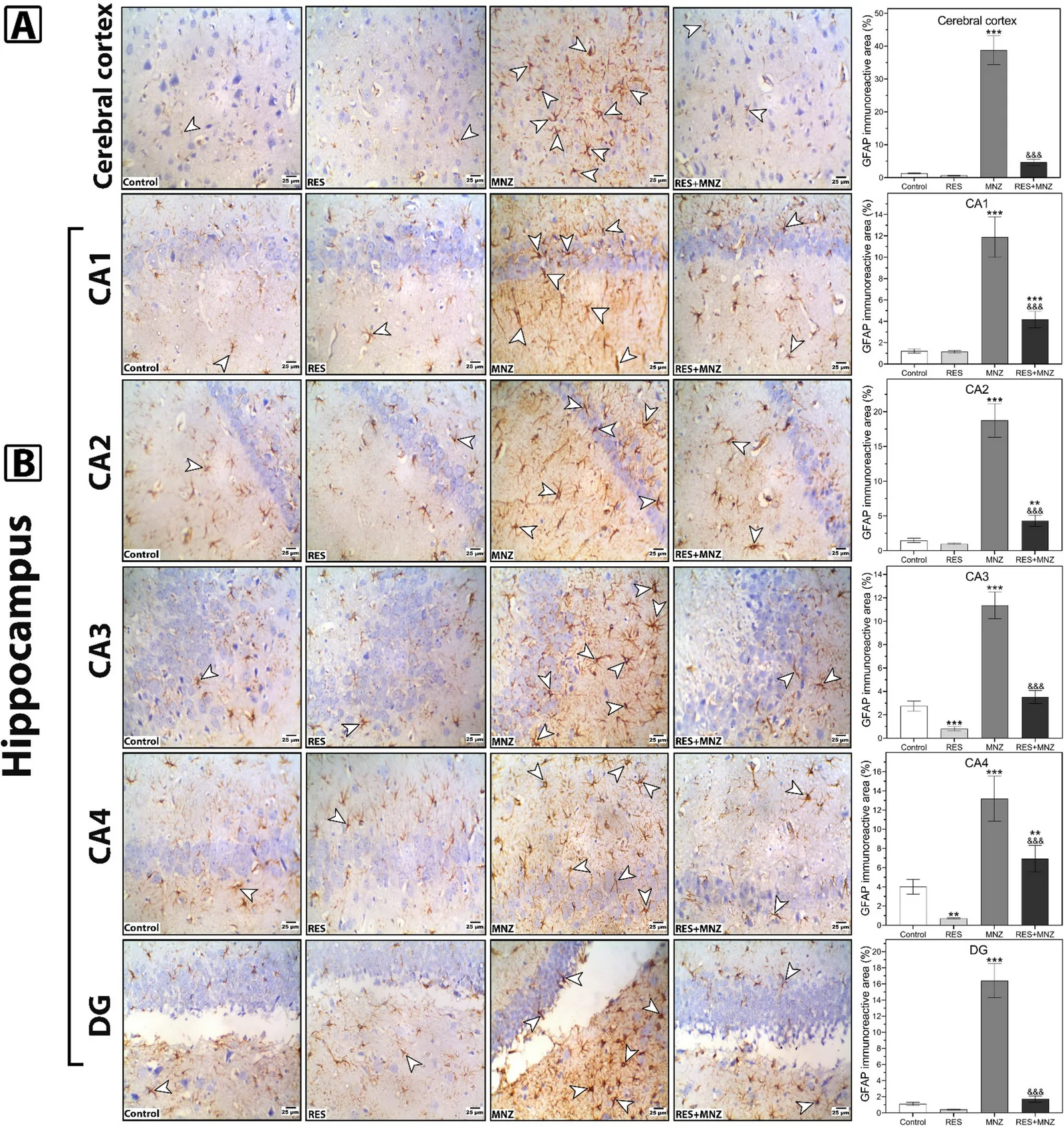
Effect of RES and MNZ on GFAP immunohistochemical expression in the cerebral cortex (**A**) and hippocampus, encompassing Cornu Ammonis (CA1, CA2, CA3, and CA4), and dentate gyrus (DG) regions (**B**) in the various studied groups of pregnant rats (X400). The control rats and RES-treated rats exhibit minimal GFAP expression within the cerebral cortex and hippocampus (arrowhead). The MNZ group shows a highly remarkable increase in GFAP immunoreactivity in the cerebral and hippocampal sections (arrowheads). RES + MNZ rats reveal moderate to near-normal GFAP expression across the examined brain areas (arrowhead). Quantitative analysis is represented as means ± SD of GFAP-immunoreactive area (%) from six microscopic fields per tissue sample. ^**^P < 0.01, ^***^P < 0.001 vs. control; ^&&&^*P* < 0.001 vs. MNZ group (Tukey’s test). RES, resveratrol; MNZ, mancozeb

The modulatory effect of RES on GFAP immunohistochemical expression in brain tissues of pregnant rats was illustrated in Fig. 5. The MNZ-treated rats showed an excessive increase (*P* < 0.001) in GFAP expression in the cerebrum and hippocampus (CA1 to CA4, and DG), reflecting severe astrogliosis compared to the control. In contrast, the RES + MNZ group exhibited a dramatic reduction (*P* < 0.001) in GFAP expression in all studied brain tissues compared to the MNZ group. Interestingly, injection of RES alone significantly (*P* < 0.001 and *P* < 0.01) decreased GFAP expression in CA3 and CA4 of the hippocampus compared with the control, as well as keeping GFAP expression in all other studied brain tissues comparable to the control.

### RES Modulated the Levels of Mitochondrial Apoptosis-Regulating Proteins in MNZ-Treated Pregnant Rat Brains

**Fig. 6:**
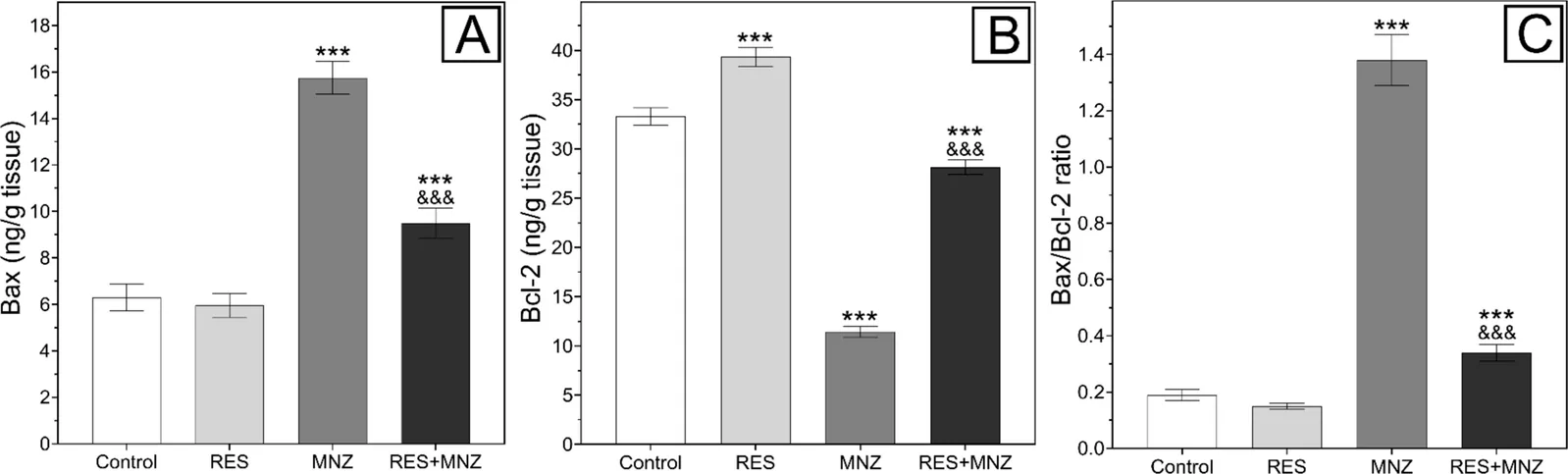
Effects of RES and MNZ on the levels of Bax (ng/g tissue; **A**), Bcl-2 (ng/g tissue; **B**), and Bax/Bcl-2 ratio (**C**) in the brain tissues of all experimental pregnant rat groups. Values are expressed as mean ± SD (*n* = 6); ^***^, ^&&&^ are significant at *P* < 0.001; ^***^ is in comparison with the control; ^&&&^ indicates comparison with the MNZ-rats (Tukey’s test). RES, resveratrol; MNZ, mancozeb

The ameliorative effect of RES injection on mitochondrial apoptotic regulatory proteins in the brain was displayed in Fig. 6. The level of the proapoptotic protein Bax and the Bax/Bcl-2 ratio increased significantly (*P* < 0.001) (Bax increased by 150% vs. the control group), (Bax/Bcl-2 ratio increased to 1.38 ± 0.09 compared to the control group 0.19 ± 0.02), whereas the level of the antiapoptotic protein Bcl-2 was reduced significantly (−65.62%, *P* < 0.001) in MNZ-treated rat brains, as compared to the control. Pretreatment with RES effectively reversed these alterations, elevating the Bcl-2 level (145.85%, *P* < 0.001), while decreasing (*P* < 0.001) the Bax level and the Bax/Bcl-2 ratio in the rat brain (−39.68% and −75.36%, respectively) compared with the MNZ rats. RES alone maintained Bax and the Bax/Bcl-2 ratio at the control level and significantly increased the Bcl-2 level compared with the control rats (18.17%, *P* < 0.001).

### RES Attenuated DNA Damage in Maternal and Fetal Brains Following MNZ Exposure

**Fig. 7:**
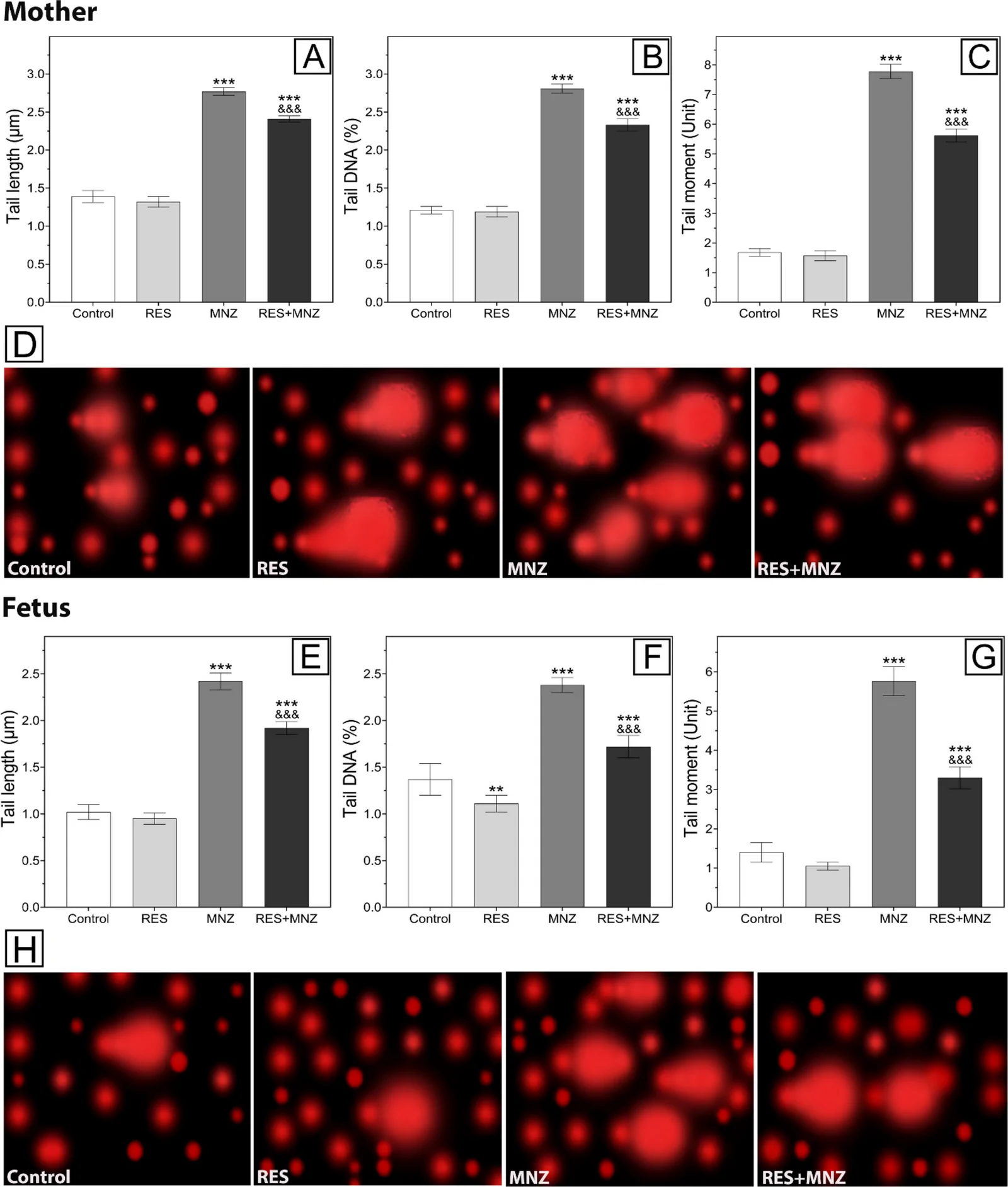
Effect of RES and MNZ on maternal and fetal brain DNA integrity on gestational day 20, assessed by the comet assay in the different studied rat groups. Parameters include maternal tail length (µm; **A**), adult tail DNA (%; **B**), maternal tail moment (unit; **C**), with representative comet photomicrographs exhibiting the effect of RES and MNZ on DNA migration in maternal brain cells (**D**), fetal tail length (µm; **E**), fetal tail DNA (%; **F**), fetal tail moment (Unit; **G**), and microscopic comet photographs revealing RES and MNZ effect on DNA migration in the fetal brain cells (**H**). Data are presented as mean ± SD (*n* = 6); ^**^P < 0.01, ^***^P < 0.001 vs. control; ^&&&^*P* < 0.001 vs. MNZ (Tukey’s test). RES, resveratrol; MNZ, mancozeb

The mitigating effect of RES on DNA damage in the brain cells of pregnant rats and their 20-day-old fetuses was evaluated by using the comet assay (Fig. 7). MNZ treatment caused a pronounced rise (*P* < 0.001) in tail length, tail DNA%, and tail moment compared to the control, indicating substantial DNA damage in both maternal and fetal brain cells. Co-administration of RES markedly reduced these elevated comet parameters (*P* < 0.001) (mother −13%, −17.08%, and −27.76%, respectively; fetus −20.66, −27.73, and −42.71%, respectively) compared to the MNZ group, demonstrating strong protection against MNZ-induced genotoxicity. In the RES-only group, fetal tail DNA% was significantly lower (−18.98%, *P* < 0.01) than control, while the remaining comet parameters were comparable to the normal values.

### RES Mitigated the Histopathological Alterations in the Frontal Cerebral Cortex and Hippocampus of MNZ-Treated Pregnant Rats

**Fig. 8:**
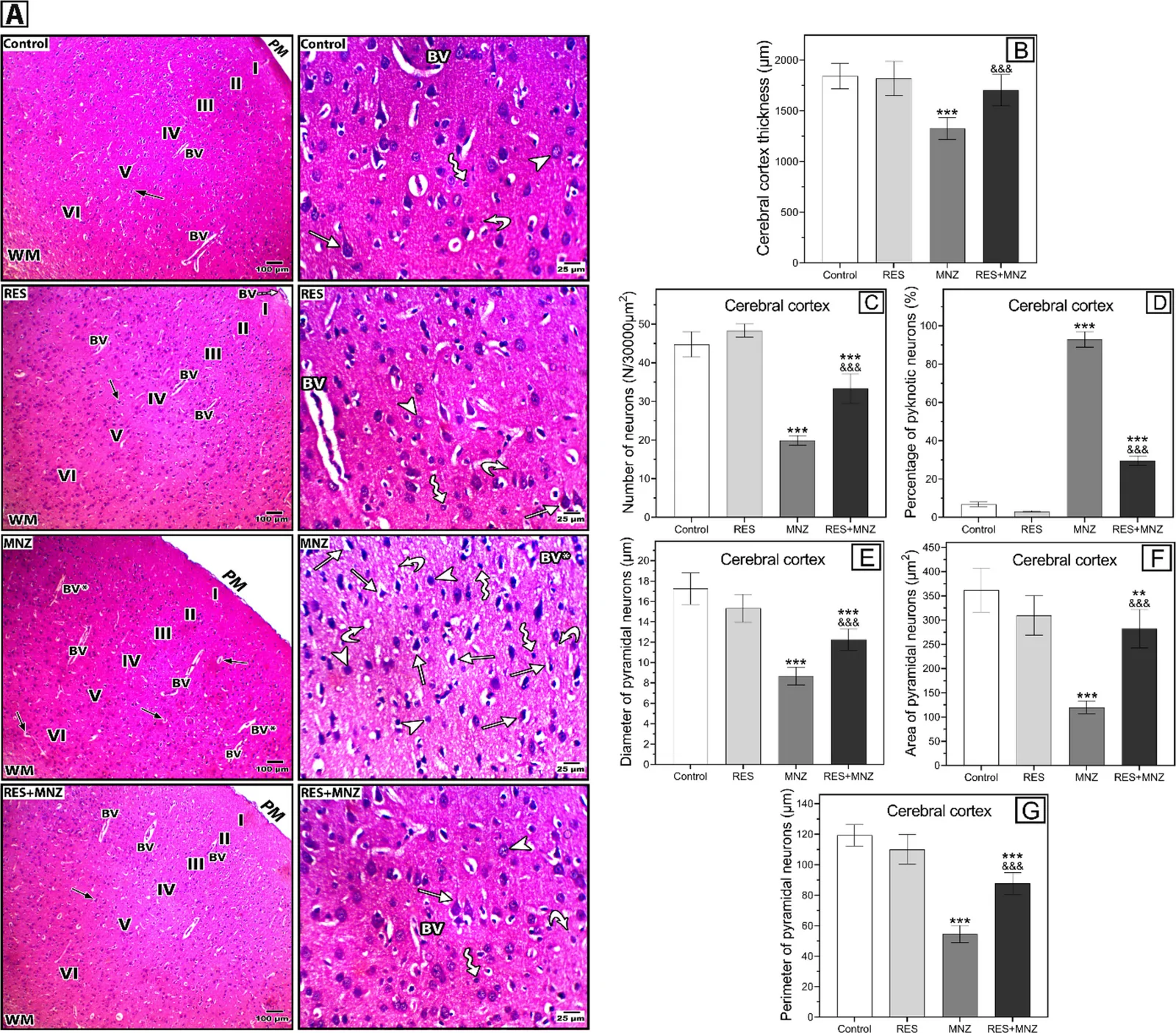
Histopathological features of frontal cerebrum in the various studied rat groups (**A**). Control and RES-treated rats show a normal six-layered cerebral cortex—molecular (I), external granular (II), external pyramidal (III), internal granular (IV), internal pyramidal (V), and polymorphic (multiform) layer (VI)—along with normal white matter (WM), healthy pia mater (PM), and many normal blood vessels (BV). Moreover, there are healthy pyramidal neurons with prominent nuclei (arrow), granular cells that have round vesicular nuclei (arrowhead), and healthy neuroglia (wavy arrow) surrounded by normal neuropils (curved arrow). MNZ-treated rats exhibit reduced cortical thickness (demonstrated in histogram B), dilated blood vessels (BV*), pyknotic pyramidal cells with darkly stained nuclei and pericellular vacuoles surrounding the shrunken pyramidal neurons (arrows), pyknotic granular neurons (arrowheads), glial pyknosis (wavy arrows), and widespread vacuolated neuropils (curved arrows). The RES + MNZ group restores normal cortical architecture, including intact cortical layers (I, II, III, IV, V, and VI), normal WM, PM, BV, and healthy pyramidal neurons (arrow), granular cells (arrowhead), and glia (wavy arrow) with normal neuropils (curved arrow). (H&E stain, X100, 400). Quantitative assessments include cortical thickness (μm; **B**), neuronal count per cortical area (N/30000μm^2^; **C**), percentage of pyknotic neurons (%; **D**), pyramidal neuron diameter (μm; **E**), pyramidal neuron area (μm^2^; **F**), and perimeter of pyramidal neurons (μm; **G**) in the control and the other studied groups. Data are presented as mean ± SD of 6 microscopic fields/tissue sample; ^**^P < 0.01;^***^P < 0.001 vs. control; ^&&&^*P* < 0.001 vs. MNZ (Tukey’s test). RES, resveratrol; MNZ, mancozeb; N, number

In both the control and RES-only groups, the cerebrum displayed normal cortical layering beneath an intact pia mater, with healthy blood vessels, followed by well-organized white matter. Moreover, the cytoarchitecture of neurons was preserved, showing normal pyramidal and granular neurons with healthy nuclei, and healthy glia that were enclosed by normal eosinophilic neuropils composed of a network of neuronal and glial processes. In contrast, MNZ exposure caused marked cortical damage, including pyknotic pyramidal, granular, and glial cells, blood vessel dilation, and neuropil vacuolation. These changes were accompanied by a significant reduction (*P* < 0.001) in cortical thickness (−28.04%), neuronal count, as well as diameter, area, and perimeter of pyramidal neurons, whereas there was a significant rise (*P* < 0.001) in the percentage of pyknotic neurons compared to the control. Co-treatment with RES markedly improved the cortical histoarchitecture, restoring healthy pyramidal and granular cells, glia, and neuropil organization. Morphometric parameters, including cerebral cortex thickness (28.45%), number of neurons, in addition to perimeter, diameter, and area of pyramidal cells, were significantly increased (*P* < 0.001), while pyknotic neuron percentage was significantly reduced (*P* < 0.001) relative to the MNZ group (Fig. 8).

Hippocampi of control and RES rats displayed the typical normal organization of CA1 to CA4 regions, including normal histoarchitecture of the proper layers, normal thickness and organized terminations of mossy fibers in the stratum lucidum layer, as well as normal polymorphic layer, pyramidal layer, and molecular layer in CA1, CA2, and CA3, and a normal single polymorphic cell layer in CA4. Healthy DG region with normal superficial molecular layer, granular cell layer, the inner polymorphic layer, the subgranular zone in between, and healthy glia. RES alone produced notable enhancements, remarkably increasing pyramidal layer thickness in CA2 and CA3 (*P* < 0.01), increasing neuronal counts in these regions (*P* < 0.01 and *P* < 0.05), whereas CA2 and CA3 pyknotic neuron percentages significantly decreased (*P* < 0.05 and *P* < 0.01) versus the control.

**Fig. 9:**
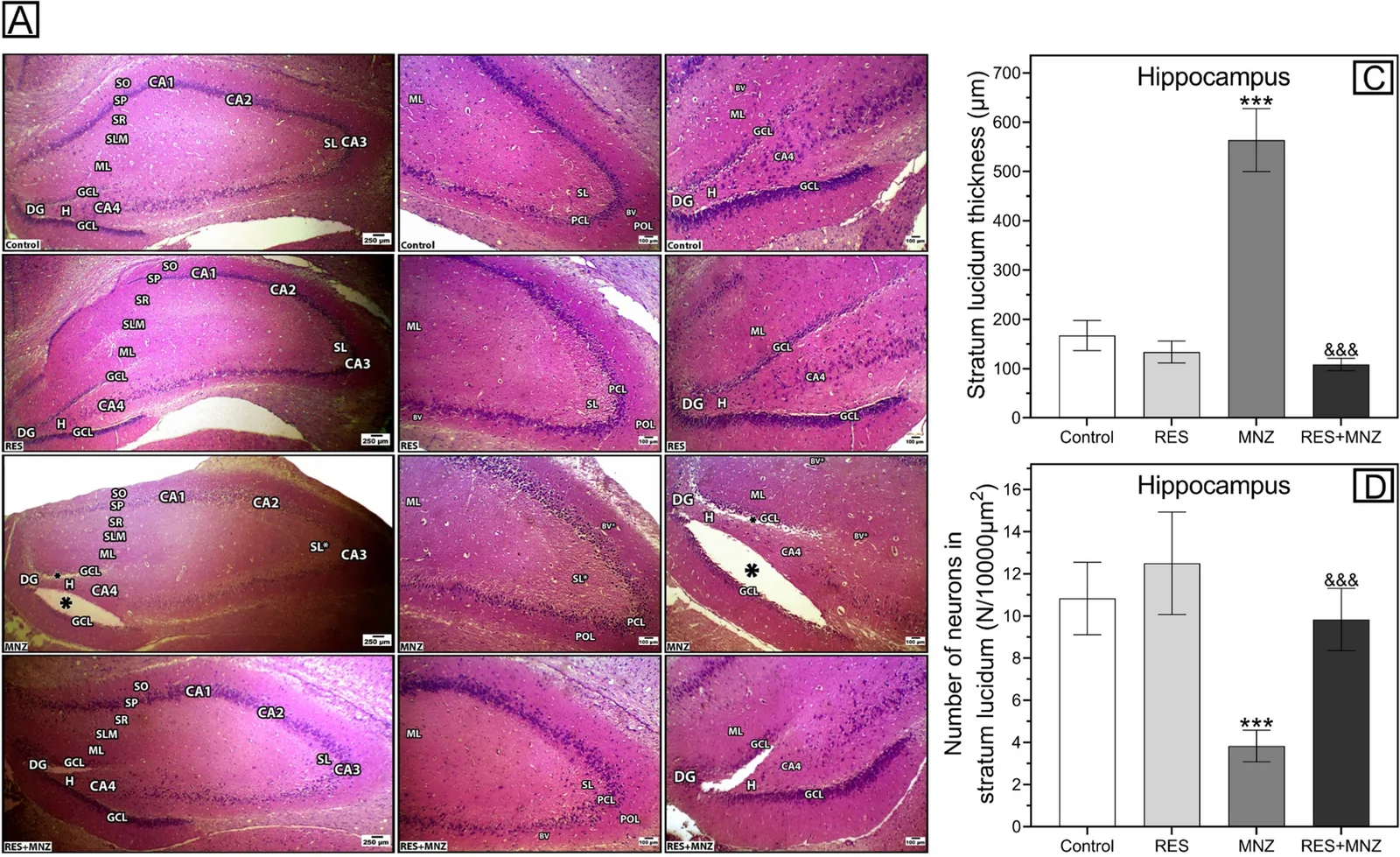

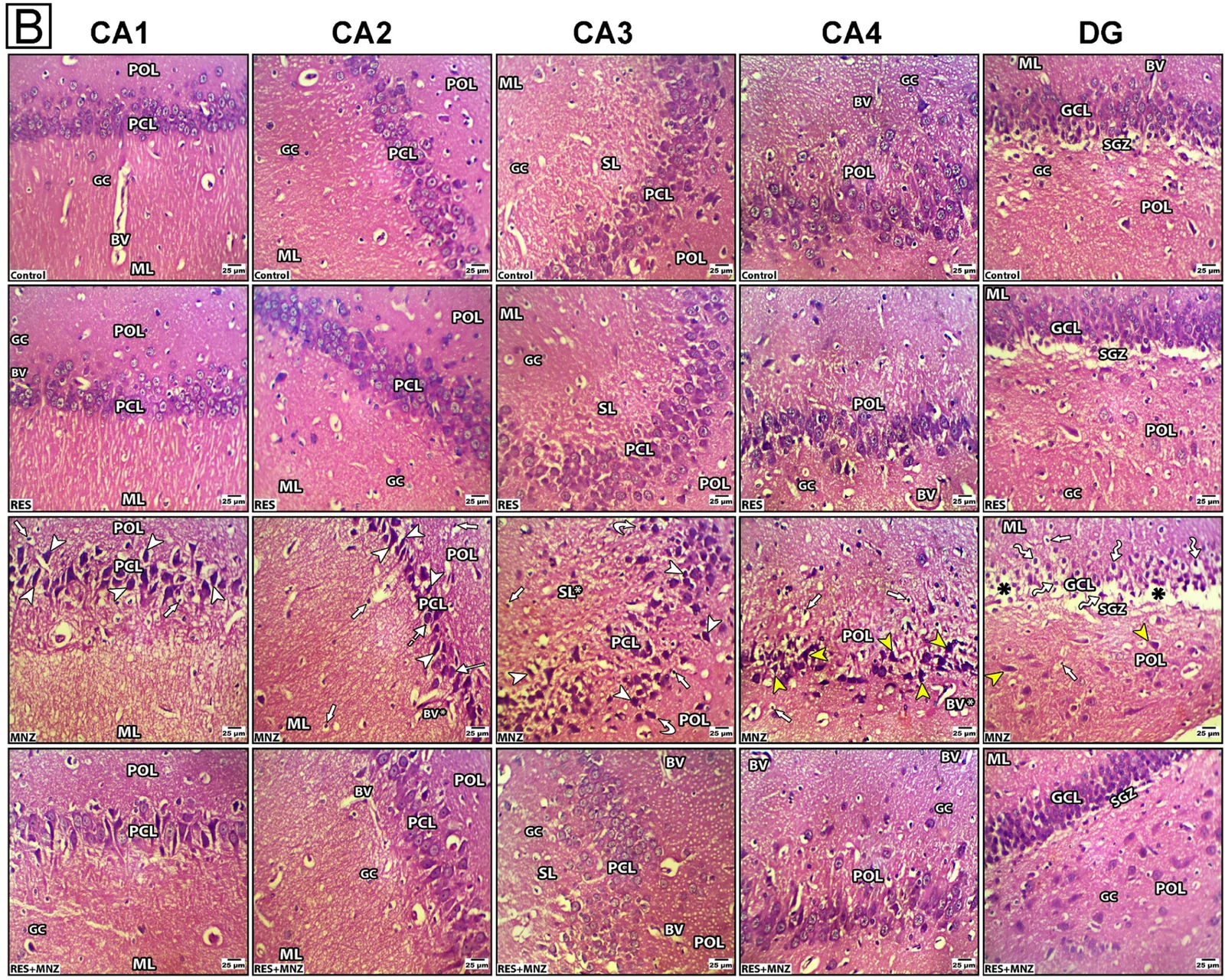

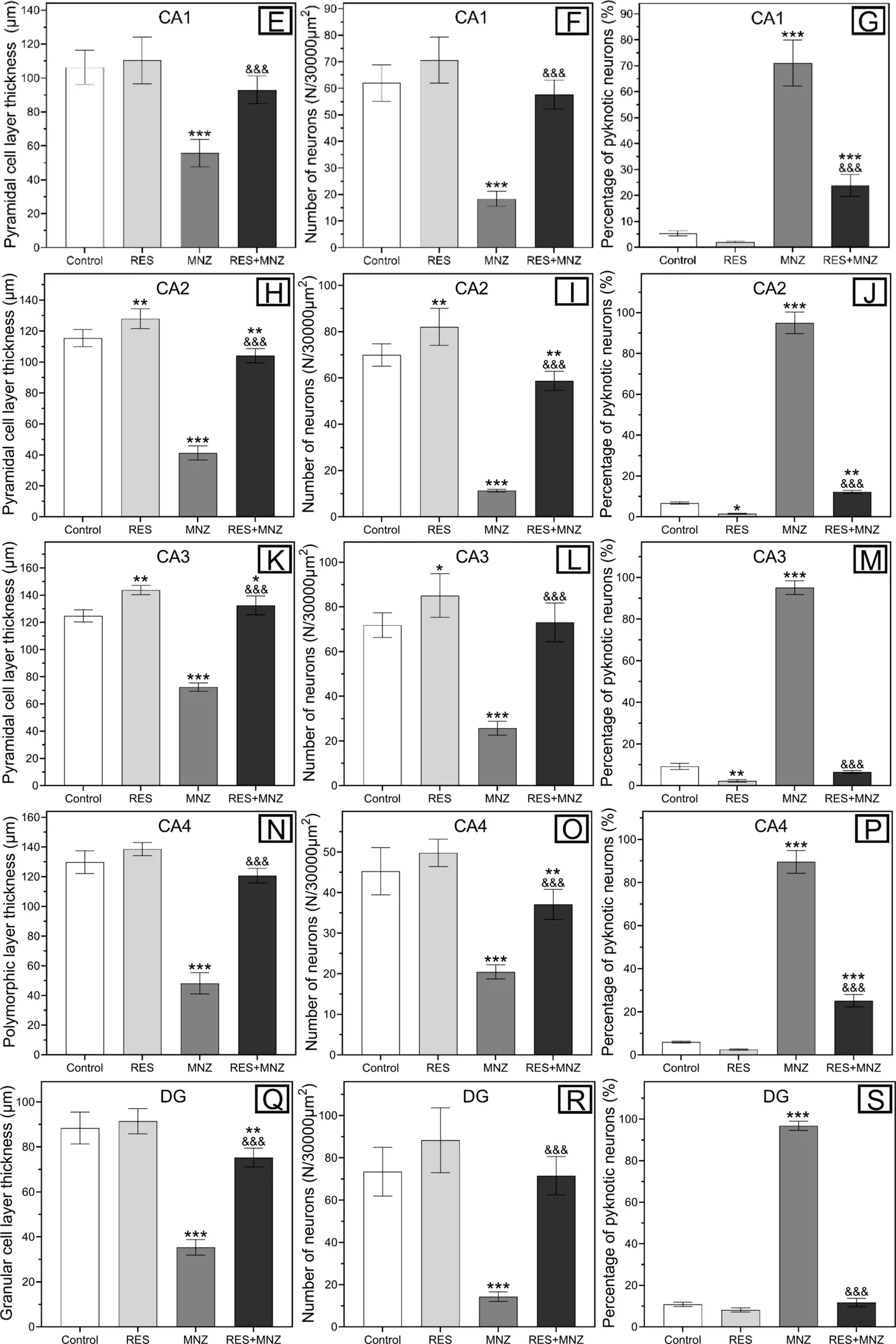
Histopathological changes in the hippocampus of the control and treated rat groups (**A**; H&E, X40 and X100), as well as higher magnified photomicrographs of the Cornu Ammonis regions (CA1, CA2, CA3, CA4), and the dentate gyrus (DG) region (**B**; H&E, X400). Control and RES-injected rats show normal hippocampal histoarchitecture of Cornu Ammonis (CA1–CA4) regions and normal proper layers: stratum oriens (SO), stratum pyramidale (SP), stratum lucidum with normal thickness and properly arranged mossy fiber terminations (SL, in CA3), stratum radiatum (SR), and stratum lacunosum moleculare (SLM). The dentate gyrus (DG) region involves a normal superficial molecular layer (ML) with intact blood vessels (BV), a well-organized granular cell layer (GCL), the inner polymorphic layer (POL), also known as hilus (H), the subgranular zone (SGZ) between GCL and POL, and healthy glial cells (GC). The CA1–CA3 contain a normal polymorphic layer (POL), pyramidal cell layer (PCL), and molecular layer (ML), while the CA4 is composed of a single polymorphic layer (POL). Mancozeb-treated rats exhibit marked pathology, including separations (asterisks) due to the neurodegeneration of the granular cells in GCL, increased thickness and disorganization of stratum lucidum (SL*), vasodilation (BV*), pyknosis and atrophy of pyramidal neurons which surrounded by pericellular haloes (white arrowheads), karyorrhexis of pyramidal cells (dashed arrow), karyolysis of pyramidal neurons (thin arrow), pyknotic glia with pericellular haloes around them (thick arrows), disorganization of PCL in CA3 (curved arrows), pyknotic mossy cells in CA4 and DG (yellow arrowheads), and many degenerated granular cells in DG (wavy arrows). In contrast, RES + MNZ rats show restored hippocampal architecture with normal neuronal and glial morphology. Quantitative measurements include: stratum lucidum thickness (μm; **C**), neuron count in stratum lucidum (N/10,000 μm^2^; **D**), CA1 pyramidal cell layer thickness (μm; **E**), CA1 neuron count (N/30000μm^2^; **F**), CA1 pyknotic neurons percentage (%; **G**), CA2 pyramidal layer thickness (μm; **H**), CA2 neuron count (N/30000μm^2^; **I**), CA2 percentage of pyknotic neurons (%; **J**), CA3 pyramidal layer thickness (μm; **K**), CA3 neuron count (N/30000μm^2^; **L**), CA3 pyknotic neurons percentage (%; **M**), CA4 polymorphic layer thickness (μm; **N**), CA4 neuron count (N/30000μm^2^; **O**), CA4 pyknotic neurons percentage (%; **P**), DG granular layer thickness (μm; **Q**), DG neuron count (N/30000μm^2^; **R**), and DG pyknotic neurons percentage (%; **S**) in groups of study. Data are means ± SD of 6 microscopic fields/tissue sample; ^*^P < 0.05, ^**^P < 0.01, and ^***^P < 0.001 vs. control; ^&&&^*P* < 0.001 vs. MNZ group (Tukey’s test). RES, resveratrol; MNZ, mancozeb; N, number

The hippocampal sections of the MNZ group showed marked structural disruption, including separations due to the degeneration of granular cells, vasodilation, necrosis of pyramidal cells, glial pyknosis, and disorganization of the pyramidal cell layer in the CA3 region. Numerous pyknotic mossy cells were observed in CA4 and the DG, along with extensive degeneration of DG granular cells. MNZ exposure significantly increased (*P* < 0.001) stratum lucidum thickness (237.19%) and percentage of pyknotic neurons across CA1, CA2, CA3, CA4, and DG regions. Conversely, neuronal counts in these regions, the thickness of pyramidal layers (CA1–CA3), the CA4 polymorphic layer, and the DG granular layer were markedly reduced (*P* < 0.001) compared with the control. Co-treatment with RES effectively restored normal hippocampal architecture, with healthy neurons and glial cells. Morphometric indices, including neuronal density in the various hippocampal regions and the thickness of the various hippocampal layers, were significantly increased (*P* < 0.001) compared with the MNZ group, while the thickness of stratum lucidum (−80.83%) and pyknotic neuron percentages (CA1–CA4 and DG) significantly decreased (*P* < 0.001) relative to the MNZ group (Fig. 9).

### Evaluation of RES Neuroprotection in MNZ-Treated Pregnant Rats and Their Fetuses by Polar Heatmap of Correlation and Pearson’s Correlation Analysis

**Fig. 10:**
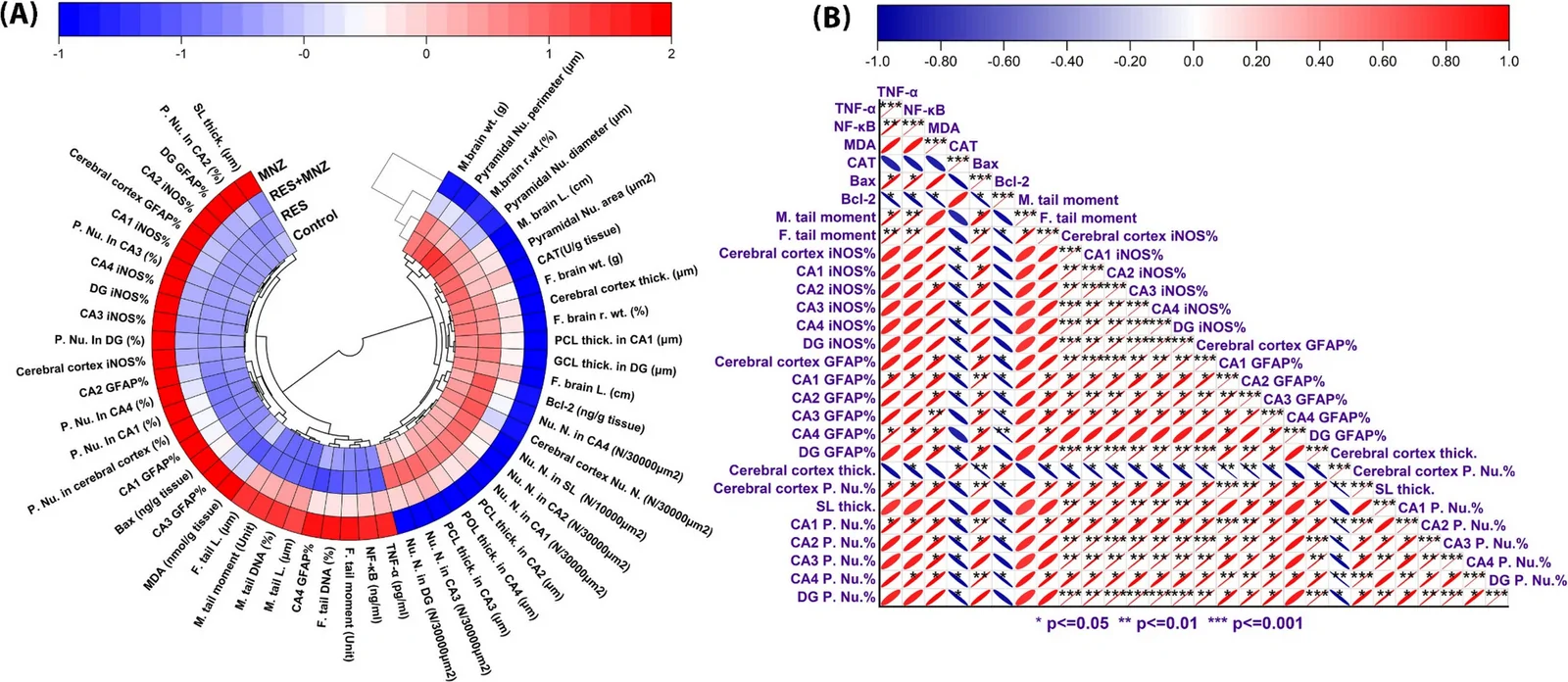
Multivariate analyses of RES and MNZ effects on maternal and fetal brains. **A** Polar heatmap of correlation displaying the mean values of all the studied parameters as colors across all experimental groups. Each cell is color-coded according to the value of the parameter’s mean in the group, as indicated in the color scale. **B** Pearson correlation matrix analysis of 28 selected parameters. Each cell represents the correlation relationship between two parameters: red ellipse bending right indicates positive correlation, while blue ellipse bending left signifies negative correlation. Ellipse width reflects the bivariate correlation; a wider ellipse refers to a weaker correlation, while a narrower ellipse indicates a stronger correlation. Significant levels ^*^P < 0.05, ^**^P < 0.01, and ^***^P < 0.001. Abbreviations: RES, resveratrol; MNZ, mancozeb; M., maternal; F., fetal; wt., weight; r. wt., relative weight; L., length; P., pyknotic; Nu., neurons; N., number; CA, cornu ammonis; DG, dentate gyrus; thick., thickness; SL, stratum lucidum; PCL, pyramidal cell layer; GCL, granular cell layer; POL, polymorphic layer

The protective effect of RES against MNZ-induced neurotoxicity in maternal and fetal brains was illustrated using a polar heatmap of correlation in Fig. 10A. Color variations reflect differences in mean parameter values across experimental groups. Control and RES groups exhibited similar parameter values, indicated by similar colors. The MNZ group values were completely antagonistic to the RES and the control values, which could be shown by strong variation in colors, reflecting severe alterations. The RES + MNZ group markedly mitigated MNZ-induced changes, with many parameter values approaching those of the control and RES groups.

Because the heatmap indicated strong relationships among the various variables, a Pearson correlation matrix of 28 selected parameters was performed (Fig. 10B). TNF-α and NF-κB serum levels, as well as iNOS expression in CA1 and CA2, GFAP expression across all studied brain regions, pyknotic neuron percentages in cerebral cortex, CA1, CA2, and CA4 in addition to stratum lucidum (SL) thickness were positively correlated (*P* < 0.01 and *P* < 0.05) with Bax level in brain tissue in addition to maternal and fetal DNA damage and negatively correlated (*P* < 0.01 and *P* < 0.05) with Bcl-2 levels in rat brain. On the other hand, the thickness of the cerebral cortex was negatively associated (*P* < 0.01 and *P* < 0.05) with Bax levels and DNA damage in the mother’s and fetuses’ brains, while positively correlated (*P* < 0.05) with Bcl-2 levels in the brain.

GFAP expression in all studied brain tissues, iNOS expression in the brain, as well as pyknotic neuron percentages correlated positively (*P* < 0.01 and *P* < 0.05) with brain MDA levels, while negatively correlated (*P* < 0.05) with brain CAT activity. Otherwise, Bcl-2 and cortical thickness correlated negatively (*P* < 0.05) with MDA levels, while positively correlated (*P* < 0.05) with CAT levels in the brain. Further, GFAP expression in various brain tissues positively correlated (*P* < 0.001, *P* < 0.01, and *P* < 0.05) with iNOS expression in various brain tissues, SL thickness, and pyknotic neurons percentage; otherwise, GFAP negatively correlated (*P* < 0.01 and *P* < 0.05) with cerebral cortex thickness. Moreover, iNOS expression in the brain positively correlated (*P* < 0.001, *P* < 0.01, and *P* < 0.05) with SL thickness and pyknotic neurons percentage, while negatively correlated (*P* < 0.05) with the cortical thickness.

## Discussion

Continuous exposure to the agricultural fungicide mancozeb (MNZ) poses notable public health concerns. MNZ has been linked to inflammation, oxidative stress, behavioral disturbances, genetic damage, and neurodegenerative outcomes with sex-dependent changes [4], affecting cognitive and memory functions [26, 27]. Resveratrol (RES), a polyphenolic compound found in grapes and peanuts, is known for its antioxidant, anti-inflammatory, and neuroprotective properties [28]. Nonetheless, it remains unknown whether RES offers a safeguard for maternal and fetal brains during the pregnancy period and the underlying mechanisms. This study added that administering RES daily before MNZ exposure in pregnant rats preserved the maternal and fetal brains and markedly improved cerebral- and hippocampal-dependent cognitive and memory function performance. These protective effects appear to result from reduced lipid peroxidation, inflammation, apoptosis, mitochondrial dysfunction, DNA damage, and histological neurotoxicity.

A recent study has recorded that pesticides such as MNZ trigger oxidative stress by elevating lipid peroxidation and diminishing antioxidant defenses [29]. MNZ-induced lipid peroxidation is thought to result from the accumulation of manganese ions (Mn^2+^) in brain tissues [4]. Elevated lipid peroxidation metabolites have been detected in the blood of almost all patients with various neurodegenerative diseases [30] and are closely linked to memory and cognition impairments [31].

In the present study, daily MNZ administration during gestation significantly increased the levels of lipid peroxidation end-product (MDA), as well as a dramatic decrease in CAT activity in the brain tissues. Conversely, maternal RES treatment improved CAT activity and lowered MDA levels in rat brains, indicating effective attenuation of oxidative damage through enhancement of the endogenous antioxidant system. These results are in parallel with a recent study that revealed that RES improved SOD, GPx, and CAT levels and reduced lipid peroxidation in mouse brains [32]. Furthermore, emerging evidence suggested that RES exerts its antioxidant action not only through free radical scavenging but also by chelating toxic heavy metals such as Mn^2^⁺ via SIRT1 activation, thereby mitigating oxidative stress [33]. Its chemical structure supports potent antioxidant and metal-chelating properties [34], which likely contribute to the observed protection against MNZ-induced lipid peroxidation.

MNZ, like many pesticides, is closely linked to persistent inflammation that can accelerate neuronal injury and contribute to neurodegenerative processes. The present study displayed a dramatic decrease in TNF-α and NF-κB levels in the sera of RES-injected pregnant rats before MNZ treatment, indicating effective suppression of proinflammatory signaling. These effects match recent studies that stated that RES decreases the IL-1β, IL-6, TNF-α, and NF-κB levels in the mouse hippocampus [5, 35]. The current study additionally recorded that RES markedly downregulated iNOS expression in the cerebral cortex and hippocampus following MNZ exposure. This agrees with evidence that RES exerts immunomodulatory effects, including inhibiting iNOS expression by controlling Toll-like receptor signaling pathways [36, 37].

The anti-inflammatory action of RES is further supported by the activation of the SIRT1 protein, which suppresses NF-κB signaling via the direct deacetylation of the RelA/p65 subunit. This deacetylation prevents NF-κB from binding DNA and initiating transcription of the key inflammatory mediators’ genes, including TNF-α, IL-6, IL-1β, and iNOS [38, 39]. Collectively, these mechanisms highlight the broad anti-inflammatory protection conferred by RES.

GFAP is a key marker of chronic astrocyte activation and plays an important role in diagnosing and monitoring neuroinflammation, neurodegeneration, and central nervous system injury [40]. Elevated GFAP levels in blood and cerebrospinal fluid are associated with progressive neurodegenerative diseases such as Parkinson’s disease and multiple sclerosis [41]. Raised plasma GFAP is particularly sensitive to early Alzheimer’s detection and correlates strongly with amyloid-β deposition and cognitive decline [42, 43]. Similarly, increased neurofilament light chain (NfL) and peripheral GFAP levels serve as early indicators of incident dementia [44]. In response to neuronal injury, NF-κB is activated, stimulating microglial release of cytokines (e.g., TNF-α and iNOS), which in turn drives chronic astrocyte activation and upregulation of GFAP expression in the brain, contributing to neurodegenerative progression [45]. These effects are affirmed by the current study’s correlation and heatmap findings that showed the positive correlation between NF-κB and TNF-α serum levels as well as iNOS and GFAP expressions in the cerebrum and hippocampus.

In the current study, MNZ exposure during pregnancy led to severe GFAP expression in the cerebral cortex and hippocampus, reflecting pronounced brain astrogliosis and neural damage. However, daily RES injection before MNZ exposure notably reduced GFAP expression in the cerebrum and hippocampal tissues, reflecting strong neuroprotective activity. These results align with Zeini et al. [46], who reported that RES remarkably attenuated the densities of astrocytes and microglia and mitigated the neuronal damage in hippocampal tissues of lipopolysaccharide-treated mice by suppressing NF-κB, IL-6, and GFAP expression. According to Rahman et al. [8], such effects arise from RES’s combined antioxidant and anti-inflammatory actions, which enhance memory, learning, and overall cognitive function, offering outstanding neuroprotection. Thus, the ability of RES to modulate inflammatory pathways and suppress GFAP overexpression during gestation provides significant protection against MNZ-induced neurodegeneration.

Neuronal apoptosis is a key feature of MNZ-induced neurodegeneration, characterized by the gradual loss of neuronal populations [47]. Previous studies validated that MNZ induces cell death and mitochondrial dysfunction in the brain through releasing Mn^2+^, which leads to elevated oxidative stress and mitochondrial changes in brain cells [4], ultimately activating intrinsic (mitochondrial-mediated) apoptosis [48]. The RES neuroprotective effect was further investigated by assessing the mitochondrial apoptotic-regulating proteins in the brain tissues of MNZ-exposed pregnant rats. In parallel with Kasaei et al. [49], the current results illustrated that RES upregulated the Bcl-2 level, which normally stabilizes the integrity of the outer mitochondrial membrane, while it downregulated the Bax level, preventing translocation to the mitochondrial membranes and formation of permeability pores, and lowered the Bax/Bcl2 ratio, reducing neuronal susceptibility to mitochondrial-mediated apoptosis, in the RES + MNZ-treated pregnant rats' brains. This shift in the Bcl-2/Bax balance inhibited the activation of the intrinsic apoptotic cascade and preserved mitochondrial integrity. Overall, these results highlight the robust anti-apoptotic and neuroprotective actions of RES against MNZ-induced neuronal injury.

RES activates the deacetylase activity of the SIRT1 protein and protects against mitochondrial ROS surge, the loss of mitochondrial membrane potential, and the ATP attenuation induced by Mn^2+^, alleviating Mn-induced mitochondrial failure mediated by DRP1 through modulating the SIRT1/PGC-1α signaling pathway [50]. Moreover, RES prevented excessive opening of the mitochondrial permeability transition pore in dopaminergic neurons, thereby maintaining mitochondrial function and improving cognitive dysfunction in the case of Parkinson's disease [11]. In addition, RES regulates critical genes involved in antioxidant defense, cellular survival, and mitochondrial dynamics, as well as neuronal protection by upregulating mitophagy through pathways such as SIRT1 and AMPK/ERK signaling pathways [51]. Thus, the antiapoptotic effects of RES offered neuroprotection against MNZ-induced apoptotic response during pregnancy.

Neurodegenerative disorders are closely linked to programmed DNA damage, which deteriorates DNA repair processes and promotes progressive neuronal loss [52]. Persistent DNA damage triggers the chronic inflammatory pathways that exacerbate neuronal injury [53]. Neuronal DNA is particularly vulnerable to oxidative stress, making oxidative imbalance a major driver of genomic instability in the brain [54]. MNZ-induced oxidative stress, primarily mediated by Mn^2^⁺ accumulation, is strongly associated with DNA damage progression [47]. In the current heatmap and correlation studies' findings, maternal brain DNA damage during gestation was positively correlated with DNA damage in the brains of fetuses. A recent study has reported that there are significant correlations between DNA damage in neonates and pregnant women [55]. Further evidence shows that MNZ and its primary metabolite, ethylene thiourea (ETU), can easily cross the placental barrier, causing DNA damage in fetal cells [56]. Early-life Mn exposure during intrauterine life impairs infant neurodevelopment, with sex-specific deficits in cognition and socio-emotional behavior [57].

Our study detected that RES injection notably protected against DNA damage in brain cells of mancozeb-exposed pregnant rats as well as their fetuses, as reflected by the remarkable decrease in the comet assay parameters compared with the maternal and fetal brains of mancozeb-treated rats. These findings align with a recent study that validated that RES maintains DNA stability against arsenic by inducing DNA repair pathways and countering oxidative genotoxicity in noncancerous cells of mammals [12]. RES supports redox homeostasis in neuronal cells by strengthening endogenous antioxidant defenses [13]. In the current study, there was a positive correlation between the studied inflammatory mediators (NF-κB, TNF-α serum levels, and iNOS expression), GFAP expression, MDA, BAX levels, and DNA damage in the brains of mothers and fetuses. In a recent study carried out by Zamanian et al. [58], RES activates the Nrf2 signaling pathway, enhancing antioxidant enzyme expression and scavenging free radicals, thereby limiting DNA oxidation, maintaining DNA integrity, and preventing neuronal cell death. In agreement with these findings, our results suggest that RES provides effective protection against mancozeb-induced neuronal DNA damage in both maternal and fetal brain tissues during gestation, preserving genomic integrity and reducing neurotoxic injury.

The frontal cerebral cortex controls executive functions, encompassing cognition, working memory, decision-making, and emotional regulation [59]. Damage to this region, particularly to pyramidal neurons, the primary excitatory cortical cells, leads to cognitive, behavioral, and neuropsychiatric disorders such as schizophrenia [60, 61]. Cortical thinning is a well-recognized early marker of neurodegenerative and psychiatric disorders, including Alzheimer’s disease, frontotemporal dementia, and Parkinson’s disease [62–65].

In the present study, gestational MNZ exposure caused marked frontal cortical thinning, neuronal degeneration, reduced pyramidal neuron dimensions, increased pyknosis, vasodilation, and vacuolation in neuropils where synaptic connections occur. In contrast, maternal RES treatment preserved cortical thickness, maintained healthy neuronal and neuropil architecture, and normalized vascular morphology, suggesting that RES has a remarkable protective effect on the frontal cerebral cortex during pregnancy against MNZ-induced neurodegeneration, hence protecting all frontal cortex functions. Our findings confirm a recent study that stated the RES neuroprotective potential on the cerebrum against cuprizone-induced demyelination via the anti-oxidative and anti-inflammatory effects [66]. These effects are supported by the current correlation findings, which confirmed that the Bcl-2 and CAT brain levels were positively correlated with the cortical thickness, while negatively correlated with cortical pyknotic neuron percentage, which might explain the RES neuroprotective effects on frontal cerebrum during pregnancy.

The hippocampus, in the temporal lobes, is chiefly associated with memory, cognition, learning, and stress response regulation. The CA1 and CA3 handle memory encoding and retrieval [67]. However, CA2 supports social memory, exhibiting unusual gene expression and cell death resistance [68]. DG and CA4 aid in free recall mode and stimulus encoding, with mossy cells modulating excitatory and inhibitory circuits; their loss is linked to temporal lobe epilepsy [69]. Pyramidal cells of CA are susceptible to seizures, stress, and similar pathological circumstances, which can lead to hippocampal neurodegeneration [67].

In MNZ-treated pregnant rats, hippocampal neurodegeneration was evident, including necrosis of pyramidal neurons, granular and mossy cells, glial damage, disorganization of the CA3 layer, reduced neuron numbers, increased pyknotic cells, disorganization of mossy fibers, and altered layer thickness. Conversely, RES administration preserved hippocampal structure, increased neuron survival, reduced pyknotic cells, restored layer thickness, and organized mossy fibers of MNZ-exposed rats, suggesting that RES has a protective potential on pyramidal neurons, granular neurons, mossy cells, and glia of the various hippocampal regions against MNZ-induced hippocampal damage during pregnancy, hence protecting memory performance and cognition.

Additionally, the current correlation findings revealed that the serum levels of TNF-α and NF-κB, iNOS and GFAP expressions, MDA, Bax, and DNA damage were positively correlated with stratum lucidum thickness and percentage of pyknotic neurons in various hippocampal regions. Our results agree with a recent study that showed that RES augments regeneration of the hippocampal tissues, prevents cell death, and protects against long-term cognitive and memory dysfunctions by diminishing oxidative stress and regulating the neuroinflammation response via SIRT1/NF-κB mediated pathways [70, 71].

## Conclusion

In conclusion, RES injection during pregnancy offers promising neuroprotective effects against low-dose MNZ-induced brain injury. These findings suggest that RES preserves brain neuroplasticity via multiple interlinked pathways. These encompass scavenging lipid peroxidation via endogenous antioxidant activation and the metal chelating capacity obtained from its unique chemical structure. Redox balance attenuated neuroinflammation via regulation of NF-κB/TNF-α/iNOS pathways that suppressed astrogliosis via reduction of GFAP overexpression and accumulation in frontal cerebral and hippocampal tissues. These effects helped in the regulation of mitochondrial apoptotic proteins and preserved maternal and fetal DNA integrity, hence offering widespread protection of neurons and their vital functions. These results are supported by enhancement in maternal and fetal brain morphometric assessments and restoration of the normal histological structure of the frontal cerebral cortex and hippocampus. The correlation and heatmap findings affirmed these results via the positive relationship between NF-κB, TNF-α, iNOS, GFAP, DNA damage, MDA, Bax, SL thickness, and pyknotic neuron percentage, while the mentioned parameters were negatively related to Bcl-2, CAT, and cortical thickness. Consequently, RES preserves brain health and functions in the MNZ-treated pregnant rat model. While these findings are promising, it is crucial to acknowledge the limitations. The behavioral tests for memory and cognition evaluation were not included, which would have offered a deeper comprehension of the neuroprotection mechanisms. Future research should analyze other brain regions to evaluate the region-specific impacts of RES, taking into consideration various neurodegenerative disorders linked to MNZ. Overall, RES is a promising supplement for neuroprotection against neurodegeneration.

## Data Availability

The raw datasets are available from the corresponding author upon request.
